# Bias‐corrected respiratory motion standard deviation from amplitude‐binned 4D‐MRI for radiotherapy planning

**DOI:** 10.1002/mp.70745

**Published:** 2026-11-14

**Authors:** Michael Ferraro, Sirisha Tadimalla, Fidel Navarro Salazar, Eireann Cosgriff, Annette Haworth, Jonathan Sykes

**Affiliations:** ^1^ Institute of Medical Physics University of Sydney Sydney NSW Australia; ^2^ Image X Institute University of Sydney Sydney NSW Australia; ^3^ Radiation Oncology Network Western Sydney Local Health District Sydney NSW Australia

**Keywords:** 4D‐MRI, mid‐ventilation, motion management, radiation therapy, standard deviation estimation

## Abstract

**Background:**

Respiratory‐resolved magnetic resonance imaging (4D‐MRI) provides superior soft‐tissue contrast for characterizing respiratory motion in radiotherapy, but its geometric fidelity depends on the reconstruction and binning strategy. As with 4D‐CT, peak‐to‐peak amplitude derived from binned 4D‐MRI is prone to underestimation. However, as acquisitions span many respiratory cycles, the standard deviation (SD) of motion, required for probability‐based planning approaches such as mid‐ventilation, may be estimated accurately, provided binning‐induced bias is corrected.

**Purpose:**

To develop and validate a motion SD estimator for equal‐probability amplitude‐binned 4D‐MRI data that corrects for systematic underestimation caused by intra‐bin motion variability.

**Methods:**

4D‐MRI data were acquired on a dynamic motion phantom driven by five patient‐derived respiratory traces. Images were reconstructed using both a standard non‐uniform fast Fourier transform (NUFFT) and the compressed sensing‐based XD‐GRASP algorithm with varying numbers (2–20) of equal‐probability respiratory amplitude bins. An SD estimator based on the law of total variance was implemented to correct for intra‐bin variance. The accuracy of peak‐to‐peak amplitude and SD was evaluated against ground‐truth physical phantom motion, with performance under deformable motion tested on a digital phantom (CoMBAT).

**Results:**

Both reconstruction methods underestimated true peak‐to‐peak motion. While the inter‐bin SD calculation also underestimated motion variability, the proposed correction substantially mitigated this bias. It yielded robust SD estimates with median residual underestimation of 0.13 mm (5 bins) and 0.11 mm (10 bins) across patient traces. In CoMBAT, the absolute bias of the total SD estimate was below 3% from five bins onward.

**Conclusions:**

The proposed estimator provides an accurate measurement of respiratory motion SD from amplitude‐binned 4D‐MRI by accounting for intra‐bin variability. This supports the reliability of 4D‐MRI for planning strategies dependent on motion probability distributions. Additionally, the confirmed underestimation of peak‐to‐peak amplitude highlights a fundamental limitation in defining internal target volumes directly from binned 4D‐MRI.

## 1 INTRODUCTION

The geometric accuracy of external beam radiation therapy for thoracic and abdominal malignancies is fundamentally challenged by respiratory‐induced organ motion.^1^ To manage this, four‐dimensional computed tomography (4D‐CT) has become the clinical standard, informing strategies such as the motion‐encompassing internal target volume (ITV).^2^ However, 4D‐CT suffers from poor soft‐tissue contrast, reliance on ionizing radiation, and a demonstrated tendency to underestimate the true range of tumor motion observed during treatment.^3^, ^4^

Four‐dimensional magnetic resonance imaging (4D‐MRI) has emerged as a compelling alternative, offering superior soft‐tissue contrast without ionizing radiation.^5^ This modality enables the translation of margin‐reduction strategies, such as the mid‐position or mid‐ventilation (MidV) concepts, to MR‐based workflows.^6^, ^7^ These planning approaches use a time‐weighted mean target position or anatomy as the planning reference and derive a planning target volume margin that accounts for respiratory motion and other planning and delivery uncertainties. To define these margins accurately, the standard deviation (SD) of the respiratory motion probability distribution must be known. In current clinical implementations in both 4D‐CT and 4D‐MRI, SD is frequently approximated from the measured peak‐to‐peak motion amplitude (e.g., $\sigma_{br}\approx 0.36A_{br}$).^8^, ^9^, ^10^ However, given the potential of 4D‐MRI to underestimate peak‐to‐peak amplitude,^11^, ^12^ relying on this approximation introduces a compound error that may compromise geometric accuracy. An inter‐bin SD approach based directly on bin‐wise target positions has also been implemented clinically for MR‐guided mid‐position lung radiotherapy planning.^13^, ^14^

The clinical utility of 4D‐MRI is contingent on the fidelity with which motion is reconstructed from highly undersampled, usually non‐Cartesian *k*‐space data. Image formation requires complex algorithms, such as the non‐uniform fast Fourier transform (NUFFT), and image quality can be enhanced by iterative, compressed sensing (CS) techniques like XD‐GRASP.^15^ The choice of reconstruction algorithm and the number of retrospective respiratory amplitude bins directly impact the geometric accuracy of the final image series. While ITV‐based planning relies on the peak‐to‐peak motion amplitude, alternative strategies like MidV planning require an accurate characterization of the motion probability distribution, specifically SD, to define appropriate margins.^9^ Calculating SD directly from binned 4D data is non‐trivial. A naive calculation of the inter‐bin SD computed from bin‐wise target position estimates inherently neglects intra‐bin variability, leading to systematic underestimation.

In this work, we focus on equal‐probability amplitude‐binned 4D‐MRI, where each reconstructed state corresponds predominantly to a displacement range rather than a phase of the breathing cycle. Equal‐probability amplitude sorting is commonly used in self‐gated 4D‐MRI, balances the available *k*‐space samples across bins, and provides the known bin probabilities required for correction from ordered bin positions.^16^ Equal‐amplitude correction instead requires self‐gating‐derived bin occupancies, while phase‐bin means cannot recover cycle‐to‐cycle variability at fixed phase. Equal‐amplitude and phase binning are therefore assessed only as secondary simulation extensions. A controlled phantom setting is particularly well suited to isolate estimator bias and reconstruction‐induced motion attenuation, because the ground‐truth motion probability distribution is known and repeatable. This enables a direct assessment of how binning, NUFFT reconstruction, and compressed‐sensing temporal regularization (XD‐GRASP) each influence recovered motion metrics, independent of contouring, deformable registration, and patient‐specific anatomical variability.

This work presents a non‐parametric estimator for the SD of motion from equal‐probability amplitude‐binned 4D‐MRI data that corrects for bias by accounting for intra‐bin variance in target position. We validate the estimator using NUFFT and XD‐GRASP reconstructions of data acquired from a dynamic motion phantom driven by patient‐derived respiratory traces. Monte Carlo simulations assess extensions to equal‐amplitude and phase binning, while CoMBAT provides a bounded evaluation under deformable thoracic motion.

## 2 METHODS

### 2.1 Phantom acquisitions and motion traces

A CIRS Model 008Z ZEUS dynamic motion phantom (CIRS/Sun Nuclear, Melbourne, Florida) was positioned near the scanner isocenter with the direction of motion parallel to the scanner bore. The phantom comprised a fillable body, representing a heterogeneous anatomical background, and a fillable cylindrical insert containing a simulated high‐contrast tumor‐shaped object. The phantom was programmed to execute one‐dimensional superior‐inferior (SI) motion driven by five distinct patient‐derived respiratory traces selected from the LARK clinical trial dataset (TROG 17.03^17^) to represent a range of clinically observed motion patterns (Figure S1).

4D‐MRI data were acquired on a 3.0 T MAGNETOM Vida MRI scanner (Siemens Healthineers, Erlangen, Germany) using a vendor‐supplied 4D‐MRI protocol based on a T1‐weighted 3D gradient‐echo sequence with a radial stack‐of‐stars *k*‐space trajectory (StarVIBE) with golden‐angle sampling. The sequence enables retrospective self‐gating of the *k*‐space data into multiple respiratory amplitude bins. Key imaging parameters were: TR/TE = 4.00/2.57 ms, flip angle = 8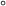, 48 partitions, for a field of view of 195 mm × 195 mm × 240 mm, yielding a resolution of 2.0 mm × 2.0 mm × 5.0 mm in the reconstructed images. Total acquisition time was 6:24 min. One acquisition used a shorter TR (2.4 ms) with the same number of acquired samples, resulting in a total acquisition time of 3:52 min. As this motion trace was non‐looped, fewer respiratory cycles were sampled. However, all quantitative results are reported as biases relative to the known input trace and aggregated across traces and bin numbers.

### 2.2 Image reconstruction

The *k*‐space data for each acquisition were reconstructed offline using two methods. First, a standard NUFFT reconstruction was performed. Second, an iterative, CS‐based XD‐GRASP reconstruction was implemented, using the NUFFT result as the initial estimate. The XD‐GRASP algorithm enforces sparsity along the temporal (respiratory motion) dimension via a total variation regularizer, with the regularization parameter λ set to 0.02, informed by the values used by Feng et al.^15^ This was selected as the strongest tested regularization that reduced streaking without the increased motion underestimation observed at higher values. For each reconstruction method, per‐acquisition image series were generated with varying numbers of equal‐probability respiratory amplitude bins, ranging from 2 to 20. Binning was performed based on a respiratory self‐gating signal extracted from the *k*‐space center along the $k_{z}$ direction, following the principal component analysis (PCA) and coil‐clustering algorithm implemented in XD‐GRASP.^15^ Radial *k*‐space spokes were sorted into equal‐count (equal‐probability) amplitude bins using the motion signal extracted from the coil cluster with the greatest power within a respiratory range of 0.1–0.5 Hz. For a five‐bin image series, the reconstruction times were 3:55 min for NUFFT and 15:53 min for XD‐GRASP. For context, the scanner inline reconstruction was also reviewed, but it did not adequately resolve motion for this phantom and was therefore not included in the quantitative analysis (Figure S2).

For one representative physical‐phantom acquisition, a sensitivity analysis compared SD‐estimation errors and reconstructed image appearance across λ=0.002–0.2 and with NUFFT (Figures S3 and S4).

### 2.3 Motion analysis

Target motion was described by the one‐dimensional displacement z of the target along the SI direction. For each reconstruction with n equal‐probability respiratory amplitude bins, a sub‐voxel template‐matching algorithm was employed to yield a target position estimate $z_{i}$ in bin i (i=1,⋯,n), ordered such that $z_{1}<z_{2}<\cdots <z_{n}$. For each series of bins, a high‐contrast region of interest of the target from the central respiratory bin was used as the template, with translations estimated by maximizing the normalized cross‐correlation and sub‐voxel refinement obtained from a three‐dimensional quadratic fit to the normalized cross‐correlation values in a 3×3×3 neighborhood of the discrete peak. Because each bin image is reconstructed from *k*‐space data assigned to that amplitude interval over many respiratory cycles, the template‐matched target position estimate $z_{i}$ is treated as an estimate of the bin‐conditional mean target position. Furthermore, as each bin contains the same fraction of time, the time‐weighted mean position coincides with the arithmetic mean of the target position estimates, $\bar{Z}=\frac{1}{n}\sum_{i=1}^{n}z_{i}$.

We consider the following SD estimators. We first include an amplitude‐derived SD estimator, widely used in clinical practice and derived under a $\sin^{6}\left(\phi \right)$ respiratory phase model:^8^

(1) $${\hat{\sigma }}^{\mathrm{amp}}=0.36\;\left(z_{n}-z_{1}\right),$$

and an uncorrected inter‐bin SD estimator, calculated as the population SD of the bin‐wise target positions and excluding intra‐bin variance,

(2) $${\hat{\sigma }}_{\mathrm{inter}}=\sqrt{\frac{1}{n}\sum_{i=1}^{n}{\left(z_{i}-\bar{Z}\right)}^{2}}.$$

We then evaluate the total SD estimator for equal‐probability amplitude bins, incorporating the proposed intra‐bin variance correction. As a non‐parametric reference, we also evaluated a quantile‐based estimator that reconstructs bin boundaries from adjacent bin‐wise target positions and integrates a piecewise‐linear quantile function. Its full definition and derivation are provided in the Supplementary Material.

#### 2.3.1 Uniform approximation (${\hat{\sigma }}^{\mathrm{total}}$)

For equal‐probability amplitude bins arising from equal‐count sorting and a smooth underlying motion distribution, we derive a closed‐form correction using the law of total variance,

(3) $$\mathrm{Var}\left(Z\right)=\underset{\text{inter-bin}}{\underset{︸}{\mathrm{Var}\left(E[Z|\mathrm{Bin}]\right)}}+\underset{\text{intra-bin}}{\underset{︸}{E\left[\mathrm{Var}(Z|\mathrm{Bin})\right]}}.$$

The inter‐bin term is estimated directly from the target position estimates,

$$\hat{\mathrm{Var}}\left(E[Z|\mathrm{Bin}]\right)=\frac{1}{n}\sum_{i=1}^{n}{\left(z_{i}-\bar{Z}\right)}^{2}.$$

Within each bin, we assume that the quantile function is approximately linear across the corresponding probability interval. Equivalently, the conditional density in z is approximately constant, giving $\mathrm{Var}\left(Z|\mathrm{Bin}=i\right)\approx w_{i}^{2}/12$ for a bin of width $w_{i}$. For smooth motion and equal‐probability bins, adjacent position gaps $g_{i}=z_{i+1}-z_{i}$ vary slowly, and the unknown widths satisfy $w_{i}\approx g_{i}$. Replacing the mean of $w_{i}^{2}$ by the mean of squared gaps yields

(4) $${\hat{\sigma }}^{\mathrm{total}}=\sqrt{{\underset{︸}{\frac{1}{n}\sum_{i=1}^{n}{\left(z_{i}-\bar{z}\right)}^{2}}}_{\text{inter-bin}}+{\underset{︸}{\frac{1}{12(n-1)}\sum_{i=1}^{n-1}{\left(z_{i+1}-z_{i}\right)}^{2}}}_{\text{intra-bin}}}.$$

Under these smoothness and equal‐probability assumptions, ${\hat{\sigma }}^{\mathrm{total}}$ provides a practical closed‐form correction requiring only the ordered target position estimates. A full derivation of this estimator is provided in Appendix A. Secondary equal‐amplitude and phase‐binning extensions are provided in the Supplementary Material.

### 2.4 Phantom evaluation of motion amplitude and SD

Peak‐to‐peak amplitude bias was evaluated for both reconstruction methods. Primary evaluation of SD‐estimator bias was performed on the NUFFT reconstructions to isolate the effect of amplitude binning from reconstruction‐induced temporal smoothing. Corresponding XD‐GRASP results at λ=0.02 were also evaluated at five and ten bins for direct numerical comparison. To validate the proposed methods, we compared the accuracy of SD estimation techniques against the ground‐truth SD calculated directly from the phantom input trace. As a baseline representing current clinical practice, we evaluated the amplitude‐derived SD estimator ${\hat{\sigma }}^{\mathrm{amp}}$ (Equation 1), based on the approximation for respiratory motion derived by van Herk et al.^8^ using a $\sin^{6}\left(\phi \right)$ model. We compared this against (1) the inter‐bin SD estimator (${\hat{\sigma }}_{\mathrm{inter}}$, Equation 2), given by the inter‐bin SD of the target position estimates, (2) the total SD estimator, ${\hat{\sigma }}^{\mathrm{total}}$, implementing the proposed uniform approximation (Equation 4), and (3) the quantile‐based reference estimator reported in the Supplementary Material. Bias was quantified across all patient traces and evaluated as a function of bin number using target positions from the reconstructed images. Supplementary Monte Carlo simulations evaluated estimator bias independently of image reconstruction and assessed extensions to equal‐amplitude and phase binning (Supplementary Methods and Tables S4 and S5).

### 2.5 Digital anthropomorphic phantom validation

The total SD estimator was additionally tested under deformable thoracic motion using CoMBAT, built on the 4D XCAT phantom^18^ and the breathing CT/MRI framework of Paganelli et al.^19^ A 20‐mm‐diameter lesion was positioned in the lower liver, and motion was driven by two concatenated traces from one LARK subject (TROG 17.03^17^). StarVIBE‐like *k*‐space data were reconstructed with NUFFT at 2 mm resolution using 2–20 equal‐probability amplitude bins. Lesion positions were estimated using the same template‐matching algorithm as for the physical phantom, with SD evaluated along the principal axis of lesion motion.

## 3 RESULTS

### 3.1 Effect of reconstruction algorithm

XD‐GRASP reconstruction produced images with lower apparent noise and fewer radial streaking artifacts than NUFFT for all acquisitions (Figure 1c).

**FIGURE 1:**
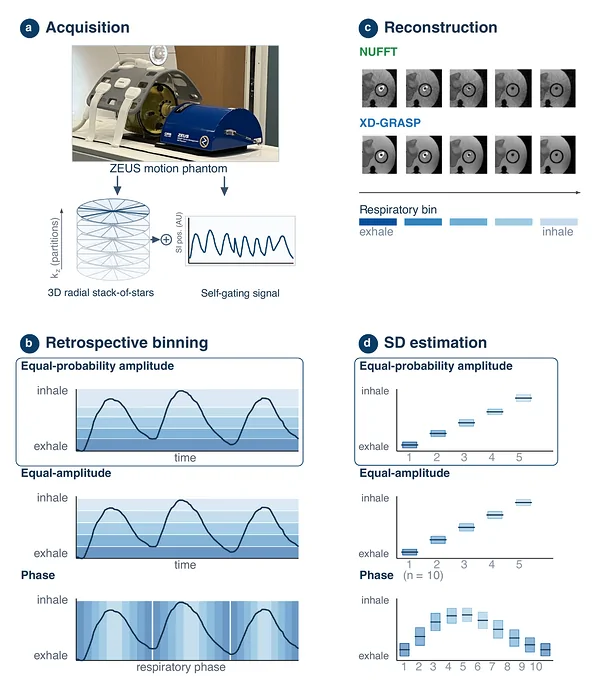
Overview of the acquisition, binning, reconstruction, and SD‐estimation pipeline. The panels show (a) the CIRS ZEUS phantom, 3D radial StarVIBE trajectory, and self‐gating signal, (b) equal‐probability amplitude, equal‐amplitude, and phase sorting of the same respiratory trace, (c) representative target images across five equal‐probability amplitude bins reconstructed using NUFFT (top) and XD‐GRASP (bottom), and (d) bin‐wise target positions and intra‐bin spread used in the variance decomposition in Equation (3). Equal‐probability amplitude binning (boxed) was used for the reconstructed‐data experiments because it provides known bin probabilities for the proposed SD estimator and distributes acquired samples equally across respiratory states.

For the representative sensitivity analysis, total SD error was similar across λ=0.002–0.02 but became increasingly negative with stronger temporal regularization (Figure S3).

### 3.2 Dependence on the number of bins

For the primary SD‐estimator analysis (Table 1), 5 and 10 amplitude‐bin reconstructions were selected as practical clinical reference settings^16^, with results reported for both NUFFT and XD‐GRASP. In NUFFT reconstructions, the total SD estimator consistently outperformed an inter‐bin SD calculation and showed reduced dependence on the number of respiratory amplitude bins relative to the amplitude‐derived SD estimator, which exhibited a marked shift from −4.40% bias at 5 bins to 7.70% at 10 bins. The quantile‐based reference estimator performed comparably but did not improve upon the total SD estimator, with median biases of −3.97% versus −3.88% at five bins and −2.25% versus −1.85% at ten bins, respectively (Table S3). Figures 2 and 3 show the effect of amplitude and SD underestimation as a function of the number of reconstructed bins, displaying only the total SD estimator alongside clinical baselines for clarity.

**TABLE 1:** Median and range (min–max) of SD‐estimator bias across the five patient‐derived motion traces for NUFFT and XD‐GRASP (λ=0.02) reconstructions, using n=5 and n=10 equal‐probability respiratory amplitude bins.

n	SD estimator	Median bias (mm)	Range (mm)	Median bias (%)	Range (%)
**NUFFT**					
5	Amplitude‐derived (Equation 1)	−0.219	[−0.282, −0.146]	−4.44	[−7.65, −2.17]
	Inter‐bin (Equation 2)	−0.197	[−0.326, −0.152]	−5.86	[−8.85, −2.41]
	Total (Equation 4)	−0.131	[−0.241, −0.036]	−3.88	[−6.54, −0.58]
10	Amplitude‐derived (Equation 1)	0.444	[0.164, 0.600]	7.70	[5.41, 14.63]
	Inter‐bin (Equation 2)	−0.125	[−0.209, −0.093]	−2.31	[−5.67, −1.71]
	Total (Equation 4)	−0.106	[−0.178, −0.066]	−1.85	[−4.85, −1.22]
**XD‐GRASP (** λ=0.02 **)**					
5	Amplitude‐derived (Equation 1)	−0.310	[−0.425, −0.207]	−6.74	[−10.22, −3.44]
	Inter‐bin (Equation 2)	−0.298	[−0.372, −0.252]	−6.25	[−10.12, −3.74]
	Total (Equation 4)	−0.192	[−0.289, −0.125]	−4.31	[−7.84, −1.85]
10	Amplitude‐derived (Equation 1)	0.435	[0.065, 0.537]	6.45	[1.03, 13.09]
	Inter‐bin (Equation 2)	−0.200	[−0.251, −0.110]	−3.78	[−6.81, −2.56]
	Total (Equation 4)	−0.183	[−0.220, −0.083]	−3.36	[−6.02, −2.04]

*Note*: Bias for the quantile‐based reference estimator in NUFFT reconstructions is reported separately in Table S3.

**FIGURE 2:**
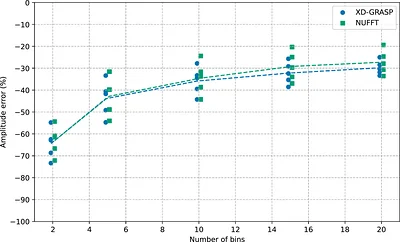
Percentage error in the peak‐to‐peak amplitude as a function of the number of respiratory amplitude bins for the XD‐GRASP reconstruction (blue circles) and NUFFT reconstruction (green squares) for five patient‐derived respiratory traces. The dashed lines show corresponding means across the five patient traces for each reconstruction method.

**FIGURE 3:**
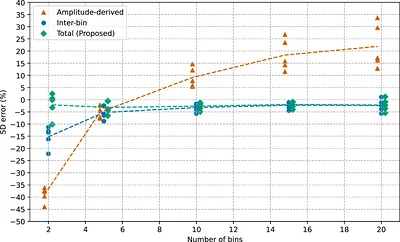
Percentage error in the SD as a function of the number of respiratory amplitude bins, showing the five patient‐derived traces for each estimator. Dashed lines represent the mean of each methodology. Amplitude‐derived SD (orange triangles), inter‐bin SD (blue circles), and total SD from Equation (4) (green diamonds) are shown for NUFFT reconstructions.

Across all five patient‐derived traces, a substantial underestimation of peak‐to‐peak amplitude is evident for both reconstruction methods (Figure 2), with a slightly greater underestimation for XD‐GRASP. This underestimation increases as the number of reconstructed bins decreases. In contrast to the amplitude case, SD can be estimated accurately with the appropriate estimator (Figure 3). We observe that the amplitude‐derived SD estimator inherits strong dependence on the number of bins linked to amplitude bias, with a cross‐over from underestimation to overestimation around n=5 bins. While the inter‐bin SD estimator also exhibits dependence on the number of bins, this bias decreases with greater bin numbers and is largely corrected even at low bin counts by the total SD estimator (Equation 4). In NUFFT reconstructions, the total SD estimator had a median bias of −1.85% at 10 bins, with performance plateauing thereafter, suggesting that 10 bins may be an appropriate choice when using the total SD estimator.

In supplementary simulations with five equal‐probability amplitude bins, total SD bias was 0.08%, 0.15%, and −2.93% for sine, uniform, and Gaussian displacement distributions, respectively (Table S4).

### 3.3 Digital anthropomorphic phantom results

In CoMBAT, the amplitude‐derived SD estimator remained strongly dependent on the number of bins and the inter‐bin SD estimator remained negatively biased (Figure 4). The absolute bias of the total SD estimate was below 3% from n=5 bins onward and remained close to zero thereafter, supporting applicability beyond rigid 1D motion.

**FIGURE 4:**
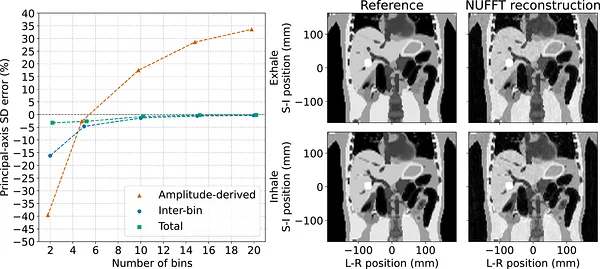
Principal‐axis SD‐estimation error as a function of the number of respiratory amplitude bins for the CoMBAT digital anthropomorphic thorax phantom. Amplitude‐derived SD, inter‐bin SD, and total SD estimates (Equations (1), (2), and 4, respectively) are shown. Coronal slices at exhale (top) and inhale (bottom) compare the reference digital phantom (left column) with the NUFFT reconstruction (right column).

## 4 DISCUSSION

This study shows that respiratory motion standard deviation (SD) can be recovered accurately from equal‐probability amplitude‐binned 4D‐MRI by correcting the inter‐bin variance for the missing intra‐bin contribution. In contrast, SD remains substantially biased when estimated from peak‐to‐peak amplitude because the reconstructed amplitude is systematically underestimated, with bin number having a greater effect on this underestimation than the modest difference between NUFFT and XD‐GRASP. Its conversion to SD additionally depends on the assumed motion waveform. The proposed correction substantially reduced the bias of inter‐bin SD estimates for both reconstruction methods, with the primary analysis based on NUFFT.

The underestimation of peak‐to‐peak amplitude, which worsened with fewer respiratory amplitude bins, is a direct consequence of temporal averaging. The outermost bins represent an average position over a time interval, which is inherently less extreme than the true instantaneous peak, especially for irregular respiratory patterns where minimal time is spent at the absolute maximum excursion. XD‐GRASP showed slightly greater amplitude attenuation than NUFFT, with a mean difference in recovered amplitude ratio of −1.9 percentage points across trace‐level averages. This suggests that while XD‐GRASP reduces streaking artifacts and may stabilize target position estimates, temporal regularization can attenuate rapid changes along the respiratory dimension and potentially smooth extreme motion states, as also observed by Mickevicius et al.^12^

This amplitude‐underestimation finding for 4D‐MRI is consistent with similar findings reported for 4D‐CT,^4^ suggesting that this is a limitation of binned 4D imaging workflows that average data over time. These findings indicate that ITV definitions derived from coarsely binned 4D‐MRI may underestimate full excursion, although the clinical magnitude of this effect would benefit from further patient and dosimetric studies.

The self‐gating waveform cannot directly replace image‐derived target amplitude because the PCA‐derived self‐gating signal is an uncalibrated surrogate with arbitrary scale and sign and may be affected by baseline drift and hysteresis. It supports occupancy and binning, but not direct recovery of peak‐to‐peak target excursion for ITV definition.

In contrast to amplitude, the SD of motion can be recovered with high fidelity. The inter‐bin SD calculated from bin‐wise target position estimates is flawed at low bin numbers, but the corrected estimator proposed in this work (Equation 4) effectively compensates for the neglected intra‐bin variance. For NUFFT, the magnitude of the median bias was below 0.3 mm across a range of realistic patient motions. For instance, the seminal van Herk margin recipe, M=2.5Σ+0.7σ, directly uses the SD of intrafractional variation to determine the necessary planning target volume (PTV) margin to ensure target coverage.^20^ Here, σ represents the combined random error term, incorporating contributions from target localization uncertainty, intrafractional instability, and respiratory motion (e.g., $\sigma_{br}$). Our findings highlight that $\sigma_{br}$ should ideally be estimated without binning‐induced bias if it is to be used reliably in such recipes. The corrected estimator proposed in this work provides a direct and robust measurement of the respiratory component $\sigma_{br}$, with application to MidV planning, where margins are typically derived from the van Herk recipe.^21^, ^22^

Current clinical margin recipes often approximate SD as $\sigma_{br}\approx 0.36A_{br}$.^10^, ^21^, ^23^ This factor arises from modeling respiratory position as $\sin^{6}\left(\phi \right)$ over phase ϕ, following Lujan et al.^8^, ^24^ For all five patient‐derived traces, $0.36A_{\text{true}}$ overestimates the true SD, indicating a mismatch between the assumed $\sin^{6}\left(\phi \right)$ waveform and the observed motion distributions (Table S1). This overestimation is present before reconstruction, with underestimation of A in the reconstructed images reducing 0.36A and partially offsetting the model error. The close agreement of 0.36A with the ground‐truth σ at n=5 amplitude bins occurs only when A is taken from the 4D‐MRI reconstruction, where the amplitude is systematically underestimated. In this specific setting (the five tested patient respiratory distributions), the Lujan‐based factor and the 4D‐MRI amplitude bias therefore appear to coincidentally partially compensate with this number of bins, an effect that is not maintained across other bin numbers or motion traces. Consequently, agreement between 0.36A and measured SD should be interpreted cautiously as it may reflect partial cancellation of model and measurement errors rather than a general relationship.

Equal‐probability amplitude binning was chosen because it balances the available *k*‐space samples across reconstructed states and provides known bin probabilities, allowing the proposed SD correction to use only ordered image‐derived target positions.^16^ Equal‐amplitude correction additionally requires self‐gating‐derived bin occupancies, which are not generally retained with reconstructed clinical images. Phase‐bin means cannot recover cycle‐to‐cycle variability at fixed phase without repeatable motion or additional surrogate or cine data.

CoMBAT results supported performance under deformable, anatomically realistic motion. The absolute bias of the total SD estimate was below 3% from five bins onward and remained close to zero thereafter. The physical‐phantom traces spanned peak‐to‐peak excursions of 13.0–30.5 mm (Table S1), although estimator performance across a broader range of excursions and lesion locations remains to be established.

The physical 4D‐MRI experiment was limited to rigid one‐dimensional phantom motion. Although the CoMBAT results supported estimator performance under deformable thoracic motion, we evaluated only one lesion and motion configuration, so broader validation across lesion locations, subject anatomies, and patient‐specific motion patterns remains necessary. The XD‐GRASP sensitivity analysis used one trace, and stronger temporal smoothing increased underestimation, warranting broader testing across motion traces and parameter choices. Equal‐amplitude and phase binning were evaluated only in simulation. Future work includes reconstruction‐level and clinical validation using a registry with pre‐treatment 4D‐MRI and corresponding orthogonal cine imaging during MR‐Linac treatment.

## 5 CONCLUSION

This work presents and validates a statistical estimator that accurately recovers the SD of respiratory motion from equal‐probability amplitude‐binned 4D‐MRI data. Our results demonstrate that peak‐to‐peak amplitude is systematically underestimated by respiratory binning, which renders ITV‐based definitions geometrically uncertain. However, probability‐based characterization of respiratory motion via SD can be performed with high fidelity from 4D‐MRI. The proposed method explicitly corrects for intra‐bin variance to yield a robust SD metric that is largely independent of the number of respiratory amplitude bins. These findings indicate that 4D‐MRI is particularly well‐suited for probability‐based margin strategies such as MidV planning. Secondary simulations supported the equal‐amplitude extension but showed that phase correction requires repeatable motion or additional within‐phase information. The CoMBAT results supported applicability under deformable motion, although further validation across lesions and patients remains necessary.

## CONFLICT OF INTEREST STATEMENT

The authors declare no conflicts of interest.

## Supporting information



Supporting Information

## APPENDIX A DERIVATION OF THE CORRECTED SD ESTIMATOR

Let Bin∈{1,⋯,n} index equal‐probability amplitude bins with P(Bin=i)=1/n. Let Z denote the one‐dimensional target position and let $z_{i}\approx E\left[Z|\mathrm{Bin}=i\right]$ denote the bin‐wise target position estimates, with $\bar{Z}=\frac{1}{n}\sum_{i=1}^{n}z_{i}$. From the law of total variance:

(A1)
$$\mathrm{Var}\left(Z\right)=\mathrm{Var}\;\left(E[Z|\mathrm{Bin}]\right)+E\;\left[\mathrm{Var}(Z|\mathrm{Bin})\right].$$

We estimate the inter‐bin term by

(A2)
$$\hat{\mathrm{Var}}\;\left(E[Z|\mathrm{Bin}]\right)=\frac{1}{n}\sum_{i=1}^{n}{\left(z_{i}-\bar{Z}\right)}^{2}.$$

For the intra‐bin term, we approximate Z∣Bin=i as uniform on an interval of width $w_{i}$, so $\mathrm{Var}\left(Z|\mathrm{Bin}=i\right)\approx w_{i}^{2}/12$ and

(A3)
$$E\;\left[\mathrm{Var}(Z|\mathrm{Bin})\right]\approx \frac{1}{12n}\sum_{i=1}^{n}w_{i}^{2}.$$

Assuming slowly varying bin widths for smooth motion, we approximate the mean squared widths by squared adjacent gaps, $\frac{1}{n}\sum_{i=1}^{n}w_{i}^{2}\approx \frac{1}{n-1}\sum_{i=1}^{n-1}{\left(z_{i+1}-z_{i}\right)}^{2}$. Combining terms yields

(A4)
$${\left({\hat{\sigma }}^{\mathrm{total}}\right)}^{2}=\frac{1}{n}\sum_{i=1}^{n}{\left(z_{i}-\bar{Z}\right)}^{2}+\frac{1}{12(n-1)}\sum_{i=1}^{n-1}{\left(z_{i+1}-z_{i}\right)}^{2},$$

and taking the square root gives Equation (4).
