# Supplementary material for: Bias‐corrected respiratory motion standard deviation from amplitude‐binned 4D‐MRI for radiotherapy planning
Source: Med Phys. 2026 Nov 14;53(11):e70745. doi: 10.1002/mp.70745 (PMC13769376; doi:10.1002/mp.70745)
Supplement: Supplementary file 1 [file MP-53-0-s001.pdf]

Supplementary

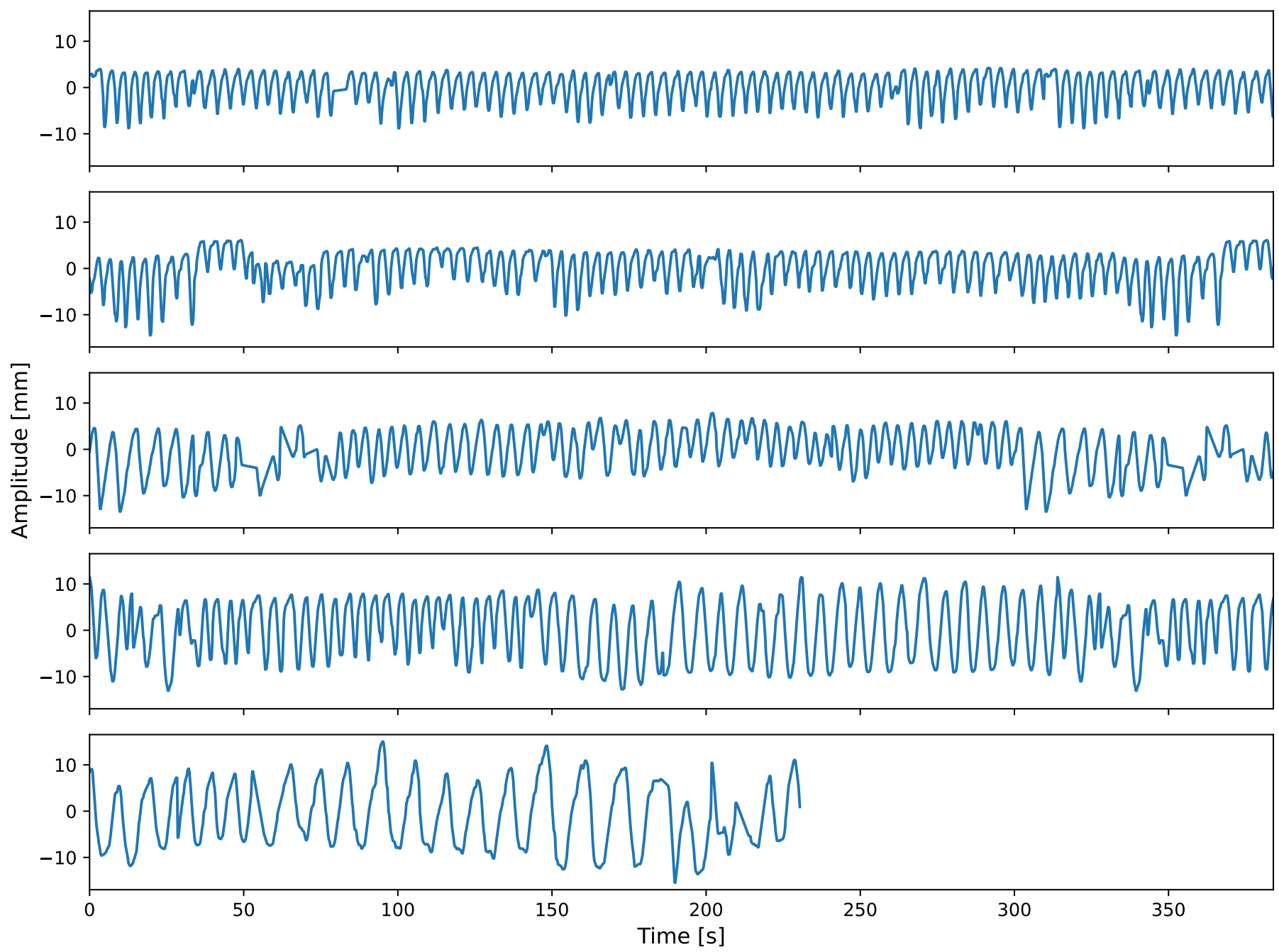

Figure S1: Patient-derived respiratory motion traces from the LARK clinical trial dataset (TROG 17.03) used to drive the phantom.

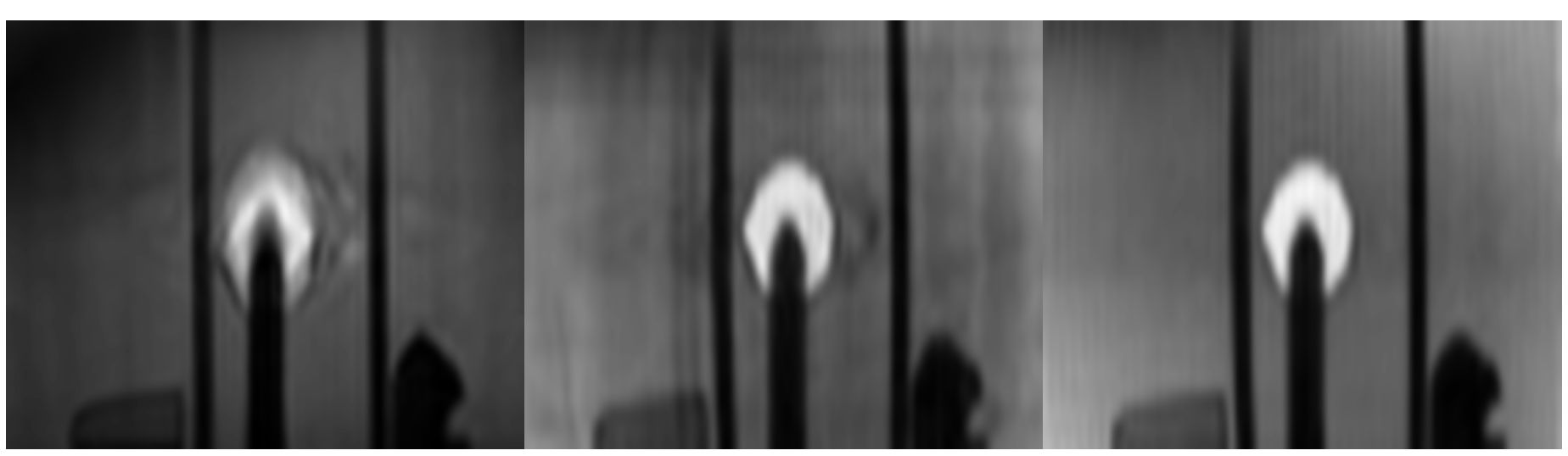

Figure S2: Example sagittal view comparing the scanner inline reconstruction (left) with offline NUFFT (middle) and XD-GRASP (right) reconstructions for bin 5 of $n = 10$ equal-probability respiratory amplitude bins.

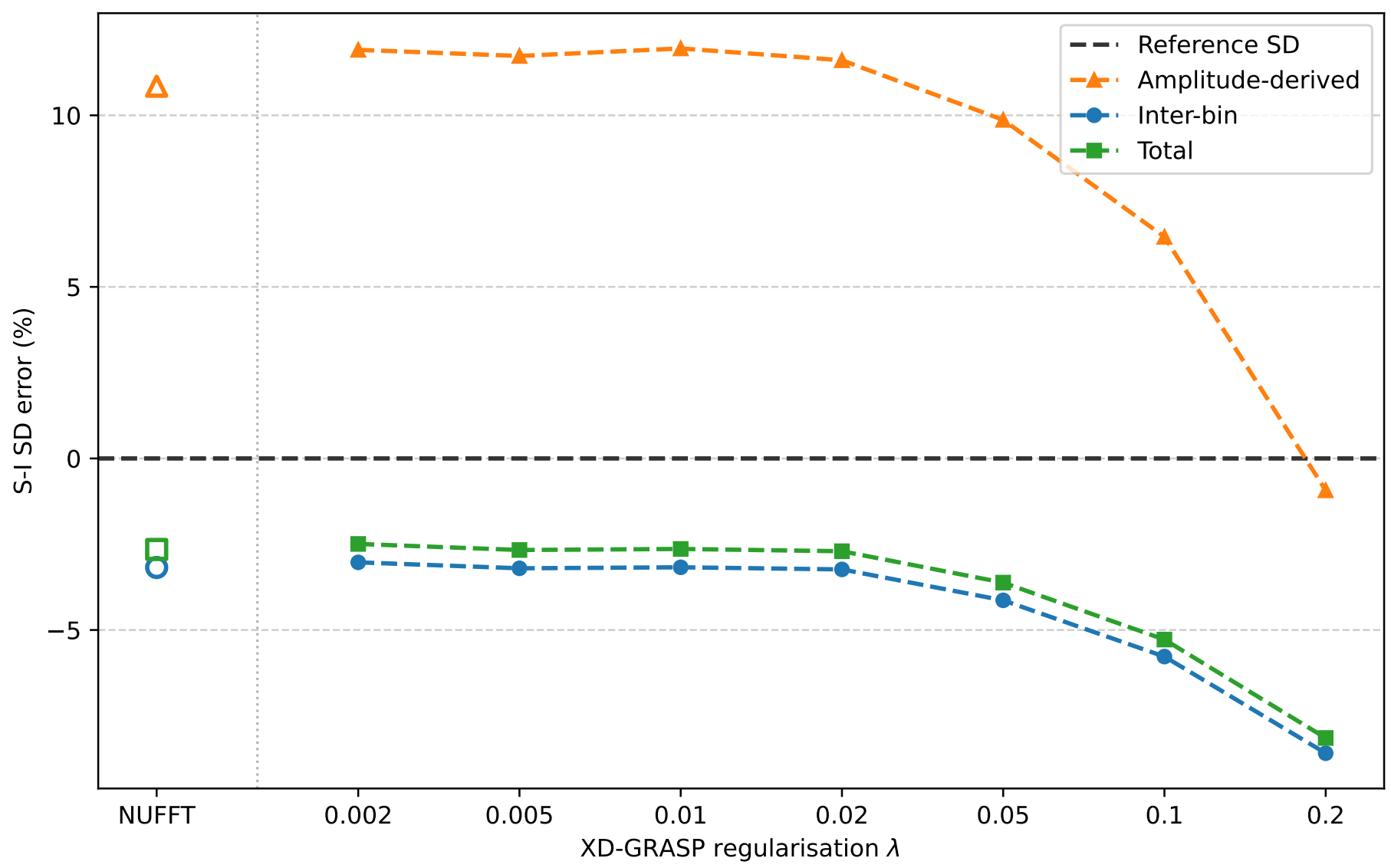

Figure S3: Superior–inferior SD-estimation error as a function of XD-GRASP temporal regularisation for one representative physical-phantom acquisition. Amplitude-derived, inter-bin, and total SD estimates are shown for $\lambda = 0.002$–0.2, with NUFFT shown for comparison. The horizontal line denotes zero error relative to the input-trace SD, and the primary analysis used $\lambda = 0.02$.

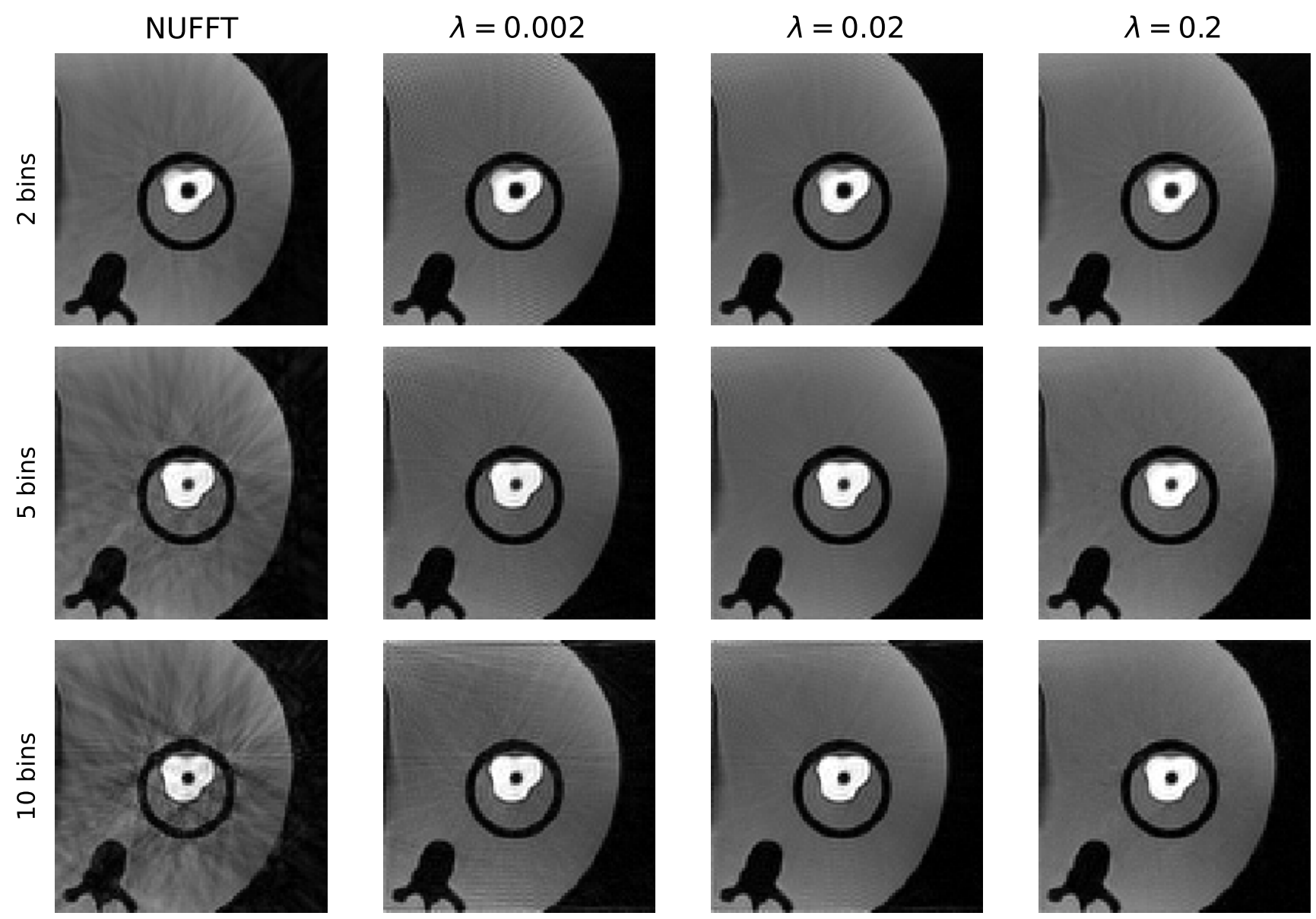

Figure S4: Representative central axial slices from a central respiratory bin of one physical-phantom acquisition reconstructed with NUFFT and with XD-GRASP using $\lambda = 0.002$, 0.02, and 0.2 for 2, 5, and 10 equal-probability respiratory amplitude bins. Visible streaking increased with the number of bins in the NUFFT reconstructions and was reduced by temporal regularisation. The primary analysis used $\lambda = 0.02$; the loss of motion fidelity with stronger regularisation is quantified in Supplementary Fig. S3.

Table S1: Summary statistics for the five patient-derived respiratory motion traces used to drive the phantom. σ_{true} is the population SD of the input displacement, $A_{\text{true}} = z_{\max} - z_{\min}$ is the peak-to-peak amplitude, and $0.36A_{\text{true}}$ is the amplitude-derived SD approximation.

Trace	σ_{true} (mm)	A_{true} (mm)	$0.36A_{\text{true}}$ (mm)
1	3.1	13.0	4.7
2	3.9	20.5	7.4
3	4.4	21.3	7.7
4	6.1	24.5	8.8
5	6.8	30.5	11.0

Quantile-based reference SD estimator

As a non-parametric reference for equal-probability amplitude bins, we also consider a quantile-based reference estimator constructed using a piecewise-linear quantile function. The bin-boundary positions $\hat{b}_j$ are inferred from the ordered bin-wise target positions as

$$\hat{b}_j = \begin{cases} z_1 - \frac{1}{2}(z_2 - z_1), & j = 0, \\ \frac{1}{2}(z_j + z_{j+1}), & j = 1, \dots, n-1, \\ z_n + \frac{1}{2}(z_n - z_{n-1}), & j = n. \end{cases}$$

On each interval $u \in [j/n, (j+1)/n]$, the inverse cumulative distribution is approximated by a linear segment joining $\hat{b}_j$ and $\hat{b}_{j+1}$. Integrating its second moment over $u \in [0,1]$ gives

$$\hat{\sigma}^{\text{quantile}} = \sqrt{\frac{1}{3n} \sum_{j=0}^{n-1} \left(\hat{b}_j^2 + \hat{b}_j \hat{b}_{j+1} + \hat{b}_{j+1}^2 \right) - \bar{Z}^2}. \qquad (1)$$

Positions were mean-centred before boundary reconstruction, so $\bar{Z} = n^{-1} \sum_{i=1}^{n} z_i = 0$. This estimator relies on known equal-probability quantile locations and is therefore reported as a reference only for equal-probability amplitude binning.

Table S2: Monte Carlo bias of the quantile-based reference estimator for equal-probability amplitude bins (5×10^5 samples, $n = 5$ bins). See Supplementary Table S4 for the amplitude-derived, inter-bin, and total estimators under the same scenarios.

Scenario	Quantile bias (%)
Sine	-2.37
Uniform	0.13
Gaussian	-0.96

Table S3: Median and range (min–max) of bias for the quantile-based reference estimator in NUFFT reconstructions of phantom acquisitions, aggregated across all patient-derived motion traces, for $n = 5$ and $n = 10$ respiratory amplitude bins. See main-text Table 1 for the amplitude-derived, inter-bin, and total estimators.

n	Median bias (mm)	Range (mm)	Median bias (%)	Range (%)
5	-0.151	$[-0.230, -0.090]$	-3.97	$[-6.26, -1.34]$
10	-0.111	$[-0.142, -0.027]$	-2.25	$[-3.49, -0.65]$

Supplementary Methods

Monte Carlo validation of SD estimators

Monte Carlo simulations with 5×10^5 samples were performed for each scenario using $n = 5$ bins. The primary comparison used equal-probability amplitude bins with sine, uniform, and Gaussian displacement distributions. Secondary simulations assessed equal-amplitude binning for the same distributions and phase binning for repeatable and variable-cycle sinusoidal motion, using the extensions defined below. Performance was quantified as the estimation bias ($100 \times \frac{\text{estimated SD}-\text{ground-truth SD}}{\text{ground-truth SD}}$).

Table S4: Monte Carlo validation of SD estimators for equal-probability amplitude binning (5×10^5 samples, $n = 5$ bins). Bias is reported as a percentage of the ground-truth SD. Corresponding quantile-based reference results are reported in Supplementary Table S2.

Distribution (Ground Truth σ)	Estimation Bias (%) Amplitude-derived SD (main-text Eq. 1)	Inter-bin SD (main-text Eq. 2)	Total SD (main-text Eq. 4)
Sine ($\sigma = 0.707$)	-4.73	-1.65	0.08
Uniform ($\sigma = 0.577$)	-0.13	-1.87	0.15
Gaussian ($\sigma = 1.000$)	0.75	-5.34	-2.93

Secondary extensions to equal-amplitude and phase binning

For a general binning variable B with bin probabilities $p_i = P(B = i)$, the variance decomposition gives

$$\hat{\sigma} = \sqrt{\sum_i \hat{p}_i (z_i - \bar{Z})^2 + \sum_i \hat{p}_i \hat{v}_i}, \qquad \bar{Z} = \sum_i \hat{p}_i z_i, \tag{2}$$

where $\hat{v}_i$ estimates residual within-bin positional variance.

For equal-amplitude bins, $\hat{p}_i$ is obtained from self-gating-derived bin occupancies and the common local width is estimated from adjacent bin means. This gives

$$\hat{\sigma}^{\text{EA}} = \sqrt{\sum_i \hat{p}_i (z_i - \bar{Z})^2 + \frac{1}{12(n-1)} \sum_{i=1}^{n-1} (z_{i+1} - z_i)^2}, \tag{3}$$

with $\bar{Z} = \sum_i \hat{p}_i z_i$. Although conceptually direct, this extension is less practical for an image-only clinical workflow because the required occupancies are not available from the reconstructed bin positions alone.

For equal-width phase bins, we evaluated a model-dependent phase-gap estimator using cyclic local widths $\tilde{w}_i = \frac{1}{2}(z_{i+1} - z_{i-1})$ in phase order,

$$\hat{\sigma}^{\text{phase}} = \sqrt{\frac{1}{n}\sum_i (z_i - \bar{Z})^2 + \frac{1}{12n}\sum_i \tilde{w}_i^2}. \tag{4}$$

This recovers finite phase-bin variance only under approximately repeatable motion. Between-cycle variability at fixed phase is not identifiable from the phase-bin means alone.

Table S5: Monte Carlo validation of the equal-amplitude and phase-binning extensions (5×10^5 samples, $n = 5$ bins). Bias is reported as a percentage of the ground-truth SD.

Scenario	Binning	Amplitude-derived SD	Inter-bin SD	Corrected extension
Sine	Equal-amplitude	-12.12	-1.42	0.15
Uniform	Equal-amplitude	-0.08	-2.02	0.00
Gaussian	Equal-amplitude	116.69	-11.29	-1.19
Repeatable sine	Phase	-9.45	-6.49	-3.03
Variable-cycle sine	Phase	-15.19	-12.41	-9.17

The equal-amplitude correction reduced inter-bin bias across the tested distributions. The phase-gap correction improved repeatable sinusoidal motion but retained -9.17% bias under variable-cycle motion.
